# Generative AI-enhanced synthetic X-ray augmentation with gradient-based selection for battery detection in WEEE

**DOI:** 10.3389/frai.2026.1881543

**Published:** 2026-08-06

**Authors:** Farhan Mahmood, George Chryssinas, Myrto Inglezou, Evangelos Katralis, Panagiotis Chatzakos, Antonios Porichis

**Affiliations:** 1 AI Innovation Centre, University of Essex, Little Abington, Cambridge, United Kingdom; 2 Tech Hive Labs (THL), Chalandri, Greece

**Keywords:** battery detection, CycleGAN, data augmentation, gradient matching, synthetic data generation, WEEE recycling, X-ray imaging, YOLO

## Abstract

Automated detection of batteries in Waste Electrical and Electronic Equipment (WEEE) using X-ray imaging is critical for safe recycling, yet collecting large annotated real-world datasets remains prohibitively expensive and hazardous. This paper proposes a three-stage synthetic data pipeline to improve battery detection under limited labeled data conditions. First, dual-energy X-ray images are generated using physics-based ray-casting in Blender with automatic pixel-level annotation. Second, the synthetic-to-real domain gap is reduced using unpaired CycleGAN-based image translation. Finally, a class-conditional gradient-alignment criterion is introduced to rank synthetic training candidates by their cosine similarity to reference gradients computed from real validation data, ensuring that only the most informative synthetic samples are injected into training. The pipeline is evaluated on a real X-ray dataset of 127 scanned WEEE devices annotated across four battery categories. Under a limited-data regime of 400 real training images, our best configuration, gradient-selected CycleGAN-translated synthetic data at +30 images per class, achieves 0.621 mAP50:95 and 0.864 mAP50, surpassing the limited-data real-only baseline (0.563/0.832) evaluated on the same held-out test set, and reaching a performance level comparable to that of a full-data reference model trained on 800 real images (0.590/0.850, evaluated on a separate test split). Ablation studies confirm that gradient-based selection consistently outperforms random sampling under matched budgets, and that domain translation provides additional complementary gains. Overall, the main outcome of this research is that adding only 120 curated synthetic images yields an absolute gain of +0.058 mAP50:95, a 10.3% relative improvement over the real-only baseline under identical training and evaluation conditions. These results demonstrate that carefully curated synthetic augmentation can compensate for real data scarcity in industrial X-ray inspection, with direct implications for scalable automated WEEE recycling.

## 1 Introduction

The rapid expansion of Waste Electrical and Electronic Equipment (WEEE) has made the development of streamlined collection and recycling systems increasingly urgent (Vermeersch et al., 2024). Within this waste stream, batteries present a particularly severe challenge, as they are laden with toxic substances that threaten human health, ecological stability, and recycling infrastructure if mishandled during disposal (Ali et al., 2022). Currently, battery removal often requires workers to manually dismantle the devices. However, the vast diversity and complexity of electronic device designs complicate this process. As a result, batteries frequently remain undetected inside the waste, introducing severe risks such as fires and explosions during subsequent processing (Terazono et al., 2024). At the same time, this manual extraction directly exposes human operators to significant safety hazards, especially when handling degraded, broken, or swollen batteries (Wu, 2026). Therefore, automated battery detection within WEEE remains highly critical.

To automate these dangerous manual processes, computer vision and Convolutional Neural Networks (CNNs) have emerged as critical enablers for intelligent waste identification and robotic recycling (Sharma et al., 2023; Wu et al., 2023). Recent advancements have focused heavily on compiling specialized real-world datasets, such as RecyBat24 (Acaro Chacón et al., 2025), and leveraging multi-modal sensory inputs to guide automated sorting systems. For instance, researchers have successfully employed visual few-shot learning fused with depth data (Keller et al., 2023), advanced 16-bit deep learning pipelines (Griffioen et al., 2024), and X-ray-based robotic fractionation (Duddek et al., 2022) to detect concealed batteries within electronic waste. However, gathering comprehensive, fully annotated real-world datasets across these diverse and hazardous industrial scenarios remains prohibitively expensive and prone to severe class imbalance. Similar difficulties have been reported in adjacent battery-related inspection tasks for instance. Riaz et al. (2025c) showed that weld defect detection in battery manufacturing is challenged by scale heterogeneity, subtle texture variation, and class imbalance, which their multi-scale convolutional model addressed to improve recall and F1-score. Moreover, practical use requires more than high accuracy. Detection models should use limited training data and work well in real inspection and robotic sorting systems (Riaz et al., 2025b). This inherent data scarcity necessitates alternative methods for data acquisition, driving the need for synthetic data generation pipelines to robustly train these automated inspection systems (Buggineni et al., 2024).

Despite these advances, two critical gaps remain unaddressed. First, existing synthetic pipelines for X-ray imaging do not model the physics of dual-energy acquisition, which is a common modality in industrial WEEE sorting systems. Second, naive injection of synthetic images into training sets frequently degrades detector performance due to domain shift and the inclusion of uninformative samples. The general question of which training samples are most informative has been studied extensively in data subset selection and active learning (Settles, 2009; Sener and Savarese, 2018; Killamsetty et al., 2021), and synthetic data curation has been explored in other domains, typically using realism or uncertainty-based filtering criteria. However, to the best of our knowledge, gradient-alignment-based selection has not previously been adapted to the synthetic-augmentation setting, and no prior work has applied task-aware synthetic sample selection to X-ray battery detection in WEEE. This paper directly addresses both gaps.

To overcome data scarcity in X-ray WEEE inspection while avoiding the pitfalls of naive synthetic augmentation, this paper makes four main contributions:

- We develop a synthetic dual-energy X-ray generation pipeline for battery-containing WEEE scenes with automatic pixel-level annotations, combining physics-based ray-casting with procedural 3D scene synthesis in Blender (Blender Online Community, 2024).
- We reduce the visual domain gap between synthetic and real X-ray images through unpaired CycleGAN-based (Zhu et al., 2017) synthetic-to-real translation, trained without requiring paired image correspondences.
- We use a class-conditional gradient-alignment criterion for selecting synthetic training samples most relevant to real validation data, adapting the GRAD-MATCH (Killamsetty et al., 2021) framework to the synthetic augmentation setting. This task-aware selection is the principal novelty of our work and, integrated with physics-based generation and domain translation, forms the first such pipeline for X-ray battery detection.
- We demonstrate that, under limited labeled data, the proposed pipeline consistently improves YOLO model (Redmon et al., 2016) battery detection performance, outperforming both a real-only baseline trained on the same data and random synthetic sampling under matched budgets, while reaching a performance level comparable to a real-only reference model trained on twice as much data.

## 2 Related work

### 2.1 Synthetic data generation

One of the most prominent applications of generative computer vision is synthetic data generation, which serves as a critical solution to the inherent data scarcity and high annotation costs found in industrial settings. Early foundational models, such as Variational Autoencoders (VAEs) (Kingma and Welling, 2013), were among the first deep learning approaches to be utilized for synthesizing training data across multiple industrial domains (Fan et al., 2023). Subsequently, the advent of Generative Adversarial Networks (GANs) (Goodfellow et al., 2014) significantly expanded the capabilities of data augmentation. Recent works have successfully implemented GAN-based synthetic image pipelines to train vision-based AI for general industrial manufacturing (Nandakumar and Eberhardt, 2025; Mahmood et al., 2025). Parallel to data-driven models, 3D computer graphics engines like Blender (Blender Online Community, 2024) have become essential for procedurally generating massive, perfectly annotated datasets. By rendering fully customized, domain-randomized 3D environments, recent studies have successfully automated the creation of annotated training data to enhance deep learning architectures (Souza et al., 2025) and combined synthetic imagery with active learning for data-efficient industrial object detection (Eversberg and Lambrecht, 2024). These automated rendering pipelines, applied to tasks such as assembly quality inspection (Zhu et al., 2025), effectively overcome the prohibitive costs and logistical barriers of manual dataset collection. Building upon these foundations, our work leverages a combined Blender (Blender Online Community, 2024) and CycleGAN (Zhu et al., 2017) pipeline to generate high-fidelity WEEE datasets.

### 2.2 Battery detection

Object detection has seen immense progress with the advent of deep learning architectures. While early two-stage detectors like Faster R-CNN (Ren et al., 2015) paved the way for high-accuracy localization, single-stage detectors, such as You Only Look Once (YOLO) (Redmon et al., 2016), have become the standard for real-time applications. YOLO represents one of the most widely used models for object detection today, as its unified architecture enables fast inference speeds, making it suitable for deployment in dynamic industrial environments (Lun et al., 2023). Building upon these industrial applications, recent works have specifically employed this technology for battery detection within WEEE using X-ray imaging. Sterkens et al. (2021) demonstrated the efficacy of deep learning for identifying and classifying various battery types in X-Ray Transmission (XRT) images of e-waste. Furthermore, recent integrated robotic solutions have leveraged AI-based object detection alongside segmentation models to fully automate the tracking and extraction of battery-containing devices using Delta robots (Giannikos et al., 2025). In parallel, recognition architectures have increasingly moved toward fusing multiple feature streams rather than relying on a single representation. For example, Riaz et al. (2025a) combined MobileNet features, Vision Transformer tokens, and handcrafted texture descriptors through cross-attention fusion to integrate local, global, and texture-aware cues for real-time recognition.

It is worth positioning the present work explicitly against the recent X-ray WEEE studies above. RecyBat24 (Acaro Chacón et al., 2025) contributes a curated real-world dataset for battery sorting, 16-bit pipelines (Griffioen et al., 2024) improve detection by exploiting the full radiometric depth of raw X-ray data, and the robotic systems of Duddek et al. (2022) and Giannikos et al. (2025) integrate detection models into physical extraction hardware. All of these directions presuppose that sufficient annotated real X-ray data are available for training. Our work addresses the complementary, data-centric question of how to train a competitive detector when annotated real data are scarce by generating, domain-translating, and selectively injecting synthetic training samples, and could in principle be combined with any of the above systems. This work utilizes a YOLO-based architecture (YOLOv8-m) to localize batteries within WEEE, retaining a standard single-stream detector so that performance differences can be attributed to the proposed data pipeline rather than architectural changes, and specifically evaluates how targeted synthetic data injection improves the mean Average Precision (mAP) of these real-world detection pipelines.

### 2.3 Data subset selection

While synthetic data generation can massively expand training sets, blindly injecting generated images often degrades model performance due to domain shifts or the introduction of uninformative, redundant samples. To mitigate this, data subset selection aims to identify the most informative training samples, thereby maximizing model accuracy while minimizing computational overhead. Early subset selection relied on isolated uncertainty heuristics, such as entropy (Settles, 2009), which often fail to capture a sample's holistic impact on the overall learning trajectory, leading to the selection of redundant samples (Kirsch et al., 2019). To address these limitations, intermediate methods evaluated data in the feature space, utilizing Principal Component Analysis (PCA) or cosine similarity between deep embeddings to construct diverse, representative core-sets (Sener and Savarese, 2018). Moving beyond feature embeddings, gradient-based selection has recently proven highly effective for deep networks by ensuring a subset's gradients closely approximate those of the target dataset. We specifically adopt the foundational GRAD-MATCH approach (Killamsetty et al., 2021) for our pipeline. This technique maximizes accuracy and efficiency by matching the gradient of the synthetic training subset with a clean, real-world validation set.

Our setting differs from the scenarios addressed by these existing selection paradigms in two important respects. Active learning selects unlabeled real samples whose annotation is expensive, so its acquisition functions are designed to minimize labeling cost, In our setting, synthetic samples arrive already perfectly labeled at negligible cost, and the binding constraint is instead the risk that domain-shifted or redundant samples harm detector training. Most existing synthetic data curation strategies filter generated samples by visual realism or distributional similarity to real data, independently of the downstream task and model state. In contrast, the gradient-alignment criterion used here evaluates each synthetic candidate by its expected effect on the detector's parameters relative to real validation data, making the selection directly task-aware. To our knowledge, gradient-matching-based selection had previously been applied to choosing subsets of real training data for efficiency, and its adaptation to curating synthetic augmentation pools, particularly for X-ray object detection, is the specific methodological gap this paper fills.

## 3 Methodology

This work evaluates whether carefully selected synthetic images can improve downstream performance under limited real-data regimes. The proposed pipeline has three main stages: (i) generating synthetic images by using 3D models designed in Blender and an X-Ray Simulator, (ii) translating synthetic images into a more realistic style using CycleGAN-based unpaired image-to-image translation, and (iii) selecting a subset of synthetic images using a gradient-alignment score inspired by gradient-matching subset selection. The selected synthetic samples are then added to the real training set for downstream object detection experiments, and compared against random synthetic selection baselines.

### 3.1 Synthetic dual-energy X-ray image generation

**Figure 1:**
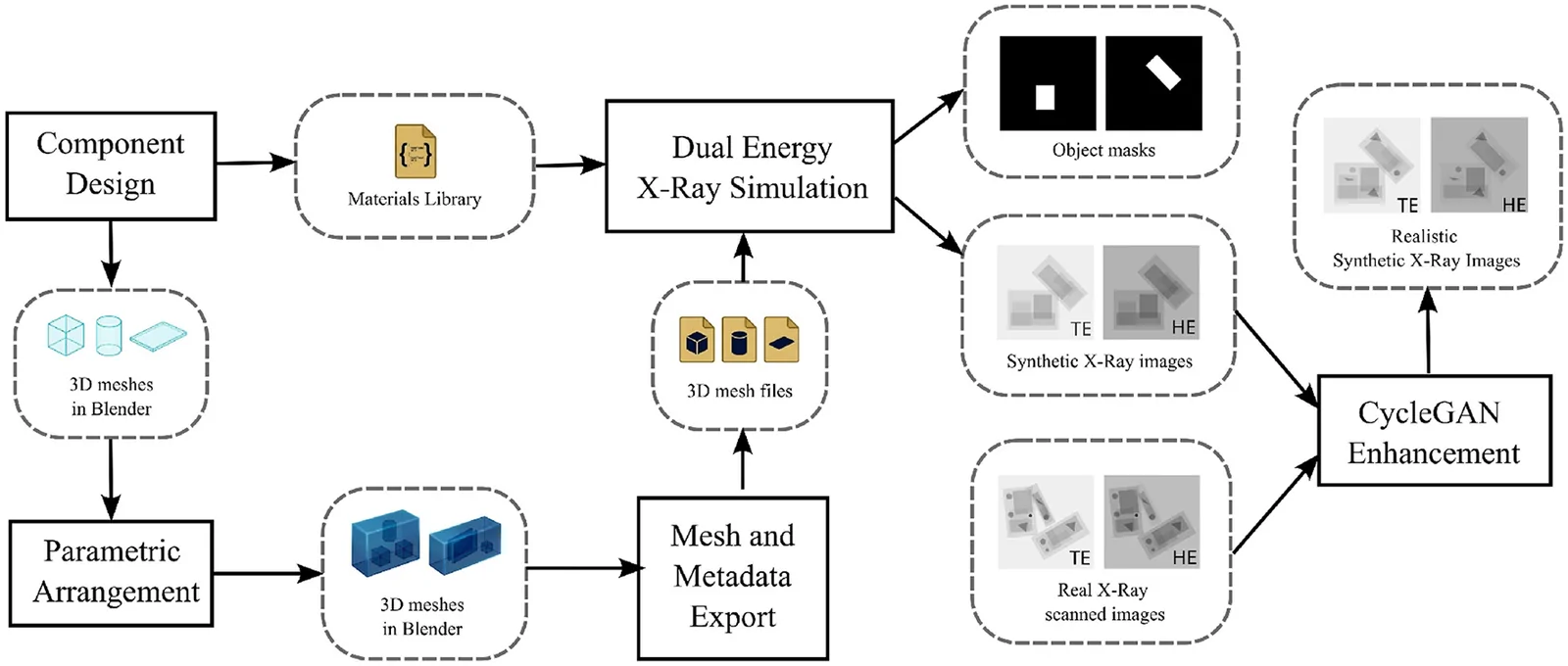
Synthetic datasets generation workflow. The workflow integrates parametric 3D asset design with a physics-based simulation engine to produce dual-energy X-ray images and corresponding pixel-accurate annotation masks. A final domain-adaptation stage using CycleGAN refines the synthetic output to match the visual characteristics of real-world scanned imagery.

The proposed synthetic data generation pipeline follows a multi-stage approach to bridge the gap between digital 3D modeling and accurate X-ray imagery. As illustrated in Figure 1, the workflow integrates parametric component design and procedural scene assembly with a physics-based simulation engine. The following sections detail the core components of this pipeline.

#### 3.1.1 Procedural 3D modeling and scene synthesis

The first step of the pipeline is to design 3D models of the WEEE. Blender proved to be a robust environment for this task due to its powerful Python API and mesh manipulation capabilities. The modeling strategy focuses on Parametric Variation and it is structured into two distinct but complementary phases.

**Figure 2:**
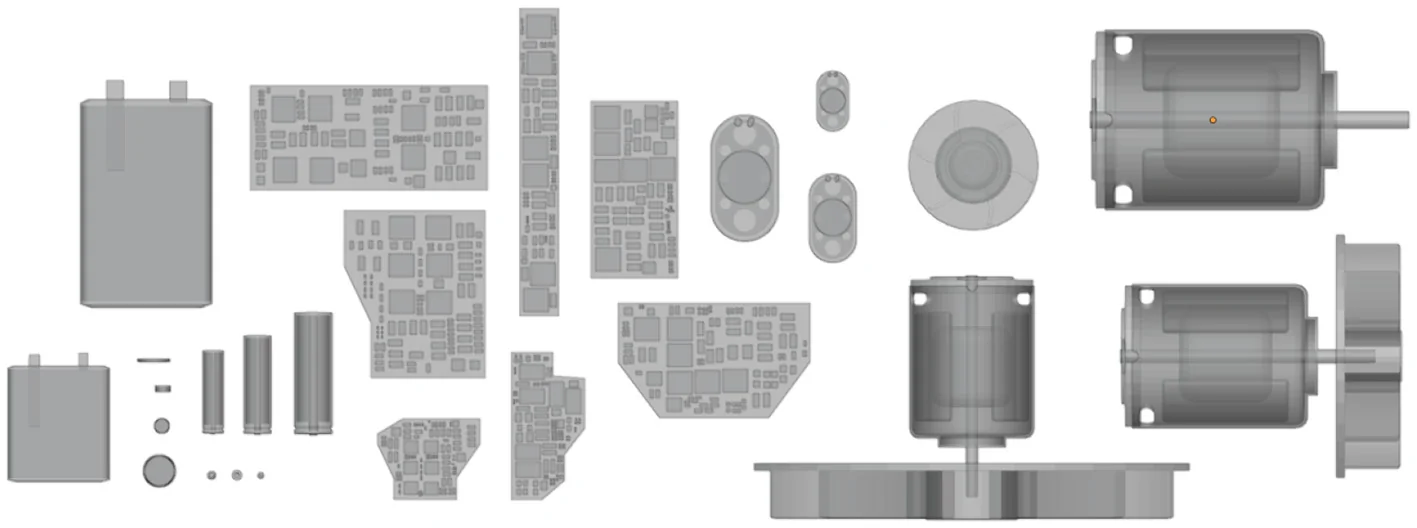
Examples of parametrically designed 3D assets for X-ray simulation. The collection includes various target battery geometries (pouch cells, cylindrical cells, and button batteries) alongside common electronic internal structures such as printed circuit boards (PCBs) and motors.

The first phase focuses on the manual design of some basic components, as shown in Figure 2. This involves creating a library of base electrical parts, such as cases, screws, circuit boards, alongside specific hazardous target items like button batteries, cylindrical and pouch cells. To enhance the diversity of the dataset, the modeling of devices with similar profiles to that of batteries, such as motors and speakers, is also included.

**Figure 3:**
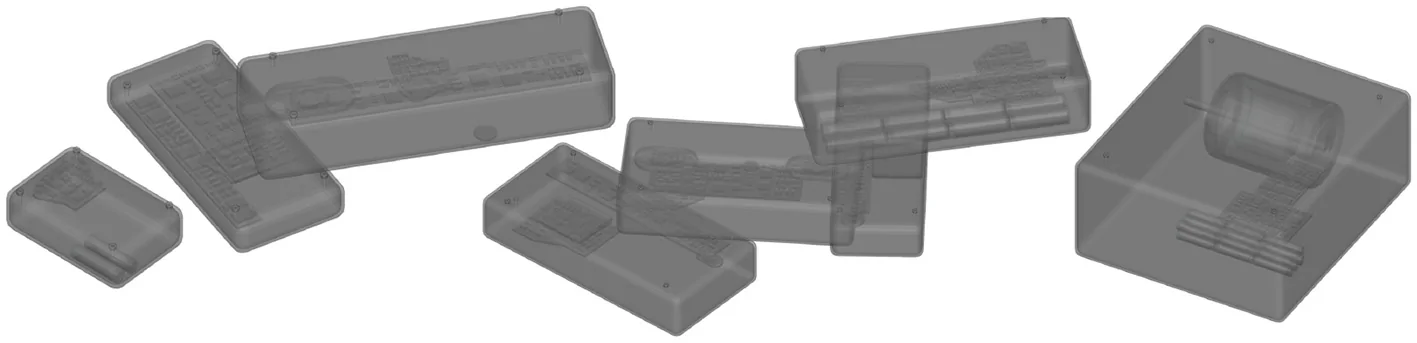
Examples of stochastically synthesized electronic assemblies and scanning scene configurations. The procedural routine applies random transformations, including varying scale, orientation, and spatial coordinates, to base components within a virtual housing.

The second phase of this process involves stochastic scene synthesis and parametric variation to generate a training dataset with significant variations. Through procedural assembly, the system programmatically modifies the base components by varying their scale, rotation, and internal placement. This methodology allows the creation of a nearly infinite variety of electrical devices from a finite set of base models. To ensure the simulation mirrors real-world conditions, the routine populates the scanning area with up to eight unique devices. This introduces significant overlap and clutter, as illustrated in Figure 3, effectively replicating the chaotic and unpredictable environment of a genuine waste stream, which is essential for training a model capable of high generalization during field deployment.

#### 3.1.2 Metadata encoding in 3D meshes

The transition from three-dimensional geometries to an X-ray image is facilitated by embedding simulation metadata within the mesh attributes. During the 3D design phase, material properties and part classifications are encoded directly into the mesh naming. These components are exported as individual STL files, allowing the simulation procedure to parse the filenames and retrieve the necessary unique identifiers that link the geometric data to a structured repository containing energy-dependent physical parameters, such as spectral attenuation coefficients. This approach ensures a strict coupling between 3D geometry and physical material properties, enabling the computational pipeline to interpret complex assemblies without manual intervention.

#### 3.1.3 Ray-casting and attenuation physics

The exported STL 3D models are loaded into the X-Ray Simulator in order to generate the Synthetic X-Rays. The core of this Simulator is a deterministic ray-casting engine that maps 3D spatial data onto a 2D image plane. For each pixel in the simulated target sensor, a primary ray is cast through the scene. The simulation calculates the final pixel intensity by modeling the interaction between these rays and the materials they traverse using the Beer-Lambert Law (Mery and Pieringer, 2021), as expressed in Equation 1:


$$\begin{array}{l} I\left(\omega \right)=I_{0}\left(\omega \right)e^{-\mu \left(\omega \right)x} \end{array}$$ (1)

where *I* is the attenuation for each energy level ω, given the initial energy of the X-ray source *I*_0_, the material attenuation coefficient μ and the distance *x* a ray traverses inside this material. This simplified model for X-Ray attenuation allowed us to obtain sufficiently detailed models without significant computational cost.

To ensure high-fidelity results, a Simulation Material library is defined. This library contains the initial spectrum of the X-ray source, *I*_0_(ω), sampled at 10 keV intervals. Each material in the simulation is defined by a unique spectral signature, providing the energy-dependent absorption coefficients μ(ω) across the simulated range. By tracking the distance an X-ray travels within each identified material, we can calculate the total attenuation for both Total Energy (TE) and High Energy (HE) outputs, mimicking the physical dual-energy detector.

#### 3.1.4 Volumetric conflict and priority resolution

A significant challenge in 3D modeling for X-ray simulation is “mesh intersection,” where independent components occupy the same spatial coordinates. In a physical environment, two solids cannot overlap; however, in 3D design, this is a common occurrence that can compromise the integrity of the distance calculation *x*. To resolve this, we introduced a Priority Assignment System into the component's mesh metadata. Every component is assigned a priority integer. During the ray-traversal process all the mesh intersections are calculated and sorted along the ray direction. By using this information, all the ray segments enclosed by a mesh are identified. If a ray occupies a region shared by multiple meshes, the algorithm identifies all active materials and calculates attenuation based exclusively on the one with the highest priority. This ensures a physically plausible simulation without requiring the design of perfectly non-overlapping CAD assemblies, significantly reducing the complexity of the 3D modeling phase.

**Figure 4:**
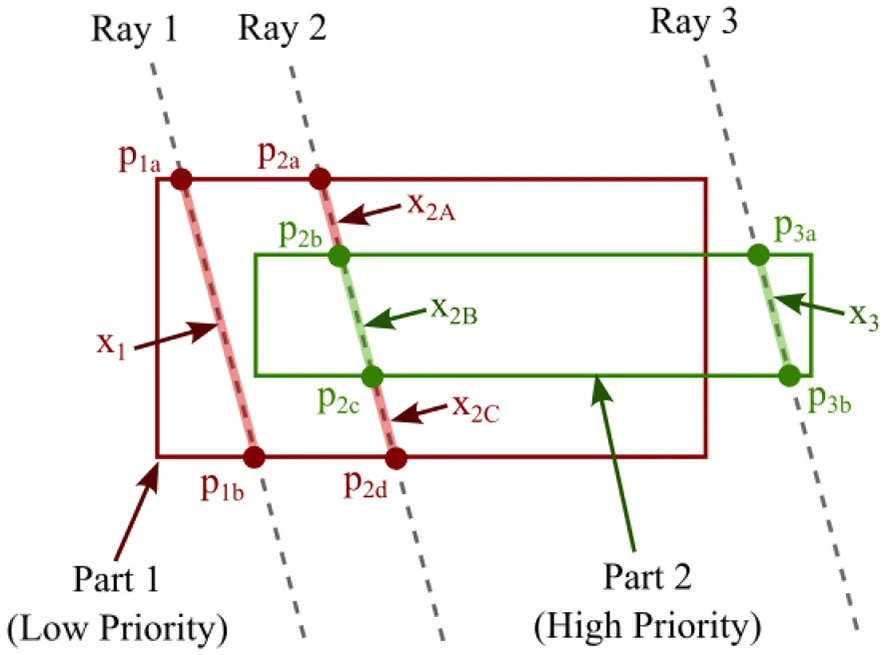
Schematic representation of the priority-based intersection algorithm for resolving volumetric mesh conflicts during ray-casting. When a simulated ray (e.g., Ray 2) occupies a region shared by multiple overlapping 3D meshes (Part 1 and Part 2), the system utilizes a priority assignment to identify active material segments (*x*_2*A*_, *x*_2*B*_, *x*_2*C*_). This ensures physically accurate attenuation calculations by prioritizing the material properties of the higher-priority mesh (Part 2) within the overlapping volume (*p*_2*b*_ to *p*_2*c*_).

For instance, when modeling a battery, the main body and the internal core may be designed as overlapping solid cylinders. Our priority system removes the need for complex boolean operations (e.g., cutting a cavity in the body for the core). By assigning the core a higher priority value, the engine ensures that the simulation calculates attenuation using the core's material properties for the overlapping volume, while the main body properties are applied elsewhere. The priority-based intersection logic is essential for handling mesh intersections where independent components share the same spatial coordinates in a 3D scene. As illustrated in the two-dimensional schematic in Figure 4, the algorithm sorts all ray-mesh intersection points, denoted as *p*, along the direction of the cast ray. For standard cases such as Rays 1 and 3, which intersect only a single component, material assignment remains straightforward. However, for Ray 2, which traverses an overlapping region, the simulator must determine three distinct segments to maintain physical plausibility. By assigning a higher priority to the internal component (Part 2), the engine correctly calculates the traversal distances: segment *x*_2*A*_ and *x*_2*C*_ are assigned attenuation based on the low-priority Part 1, while the middle segment *x*_2*B*_ is calculated based on the high-priority material properties of Part 2.

#### 3.1.5 Automated annotation and mask generation

One of the primary advantages of this synthetic pipeline is the elimination of manual labeling. Since the simulation engine identifies the specific material and part type at every intersection point, it possesses perfect knowledge of the scene's composition. By mapping the Component ID to each ray, the system generates an annotation mask simultaneously with the X-ray image. These masks provide pixel-level segmentation for batteries and other critical components. We subsequently process these masks, automatically deriving bounding boxes and ground-truth labels. This enables the rapid generation of thousands of labeled images, ready for supervised machine learning training with zero human intervention.

#### 3.1.6 Synthetic-to-real translation using CycleGAN

**Figure 5:**
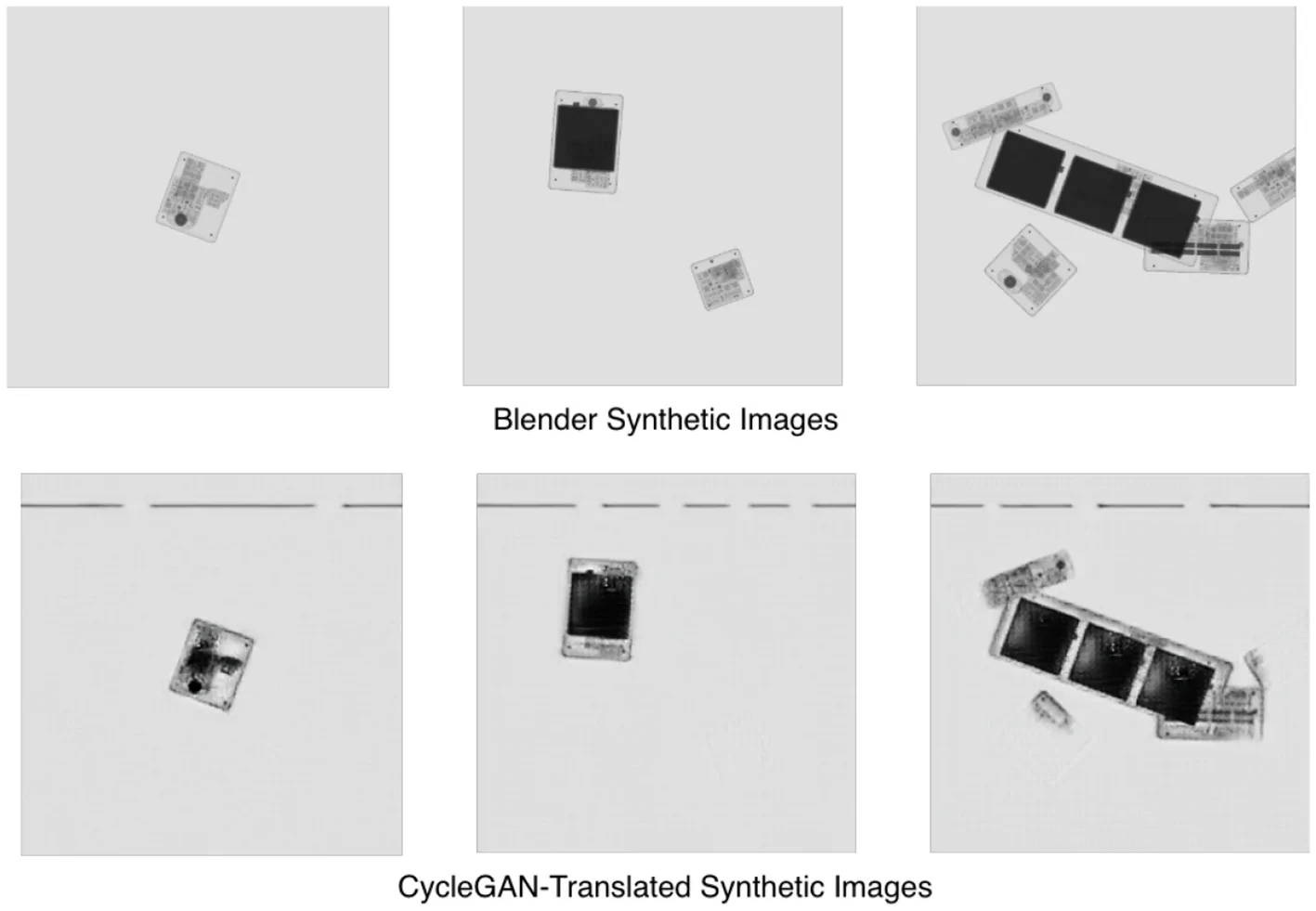
Qualitative comparison of Blender-rendered and CycleGAN-translated synthetic X-ray images.

To reduce the visual domain gap between Blender generated synthetic images and real images, we trained an unpaired image-to-image translation model based on CycleGAN (Zhu et al., 2017), which learns mappings between two domains without requiring paired correspondences. CycleGAN optimizes two generators (synthetic to real and real to synthetic) with adversarial losses, coupled with a cycle consistency objective that encourages reconstruction of the input. Qualitative examples of Blender-rendered synthetic images and their CycleGAN-translated counterparts are shown in Figure 5.

We formed two unpaired image collections:

- Synthetic domain (S): 715 Blender-generated synthetic images used as the source distribution for synthetic to real translation.
- Real domain (R): 715 real X-ray images used as the target distribution from Section 4.1.1.

The real-domain collection was drawn exclusively from the training and validation partitions of the real dataset described in Section 4.1.1. No images from either held-out real test set were included in the CycleGAN target domain. Because CycleGAN training is unpaired and unsupervised, including held-out test images could nevertheless leak transductive information about the test distribution into the translated synthetic pool, the strict exclusion of all test images avoids this risk. As dataset splits were performed at the device level (Section 4.1.1, no device contributing images to a test set contributed images to CycleGAN training either.

We followed the standard CycleGAN optimization recipe used in the literature. The model learns two mappings, *G*:*S*→*R* and *F*:*R*→*S*, trained with adversarial objectives and a cycle-consistency constraint to preserve content under round-trip translation; this enables learning without paired images. Training used the Adam optimizer. For the adversarial component we used the least-squares GAN (LSGAN) objective, which is commonly adopted to improve training stability and gradient behavior in GANs.

Because the pixel-level masks and bounding boxes are generated by the simulation engine before translation and are reused unchanged for the translated images, it is important that translation does not alter the spatial structure of annotated objects. CycleGAN can in principle introduce local shape distortions, although its cycle-consistency constraint penalizes changes that cannot be inverted and therefore discourages geometric modification of scene content. To confirm that annotations remained valid after translation, we visually inspected translated synthetic images against their Blender-rendered sources with the automatically generated annotations overlaid. Across the inspected images, translation altered appearance characteristics such as texture, contrast, and noise statistics while leaving object positions, scales, and boundaries intact, and we observed no cases in which the pre-translation bounding boxes no longer enclosed their objects. The qualitative examples in Figure 5 illustrate this behavior, with translated outputs remaining spatially aligned with their rendered counterparts.

### 3.2 Gradient-based data selection

To make synthetic augmentation effective for limited-real-data, synthetic images are not added uniformly at random. Synthetic samples are ranked and selected according to how well each sample's training signal matches the signal observed on real data. Each synthetic image is scored by how closely its loss gradient on the model's trainable head aligns with a reference gradient computed on real validation data from the same class, using cosine similarity as the alignment measure. The intuition is that if updating the model on a particular synthetic sample would move the parameters in a direction similar to the direction encouraged by real validation data, then that synthetic sample is more likely to provide useful generalization improvements than a synthetic sample whose gradient pushes the model in a conflicting direction.

For each class *c*, a reference gradient *g*_*c*_ is first computed using a small batch of real validation images of class *c*. Next, for every synthetic candidate image *i* labeled as class *c*, its gradient *g*_*i*_ is computed with respect to the model's trainable head parameters. In both cases, gradients are computed from the complete composite YOLOv8 detection objective, that is, the weighted sum of the Complete Intersection over Union (CIoU) based bounding-box regression loss, the binary cross-entropy (BCE) classification loss, and the distribution focal loss (DFL) used for box distribution regression, with the default Ultralytics loss weights. Using the full detection loss rather than the classification term alone ensures that the alignment score captures both localization and classification informativeness of each candidate. Each synthetic image is scored using the non-negative clipped cosine similarity defined in Equation 2:


$$\begin{array}{l} \text{align}\left(i\right)=\max\left(0,\cos\left(g_{i},g_{c}\right)\right). \end{array}$$ (2)

Synthetic images within each class are ranked by align(*i*) and then the top-*K* samples per class are selected for augmentation. This approach is motivated by the broader idea of gradient matching for subset selection, where one seeks a subset whose gradients approximate gradients of a larger reference set (training or validation), as explored in GRAD-MATCH (Killamsetty et al., 2021).

Computing gradients over all model parameters can be expensive, particularly for deep detectors. To keep selection lightweight and directly tied to class-discriminative updates, we compute gradients only with respect to the model's trainable detection head. This choice reduces computation while preserving sensitivity to the updates that govern downstream detection performance. The resulting ranked list is used to select the top-*K* synthetic images per class for downstream detector training.

## 4 Experiments

The experimental evaluation was designed to test whether carefully selected synthetic images can compensate for limited labeled real data in X-ray battery detection. Consistent with the methodology introduced in Section 3, all experiments followed the same three-stage pipeline and were conducted using the same YOLOv8m detector, motivated by the suitability of YOLO style detectors for real-time industrial localization and by prior work on battery detection in X-ray and WEEE settings (Redmon et al., 2016; Lin et al., 2023; Sterkens et al., 2021). The experiments evaluate three questions, whether synthetic augmentation improves low-data YOLOv8m battery detection, whether gradient-based selection outperforms random synthetic sampling under matched budgets, and whether CycleGAN translation provides additional benefit over raw Blender synthetic images.

### 4.1 Dataset

For the object-detection study, we used a balanced experimental protocol centered on a low real-data regime. For the full-data reference regime, we used 800 real training images and evaluated on a 117-image real test set. For the limited-data regime, we used 400 real training images, or 100 images per category, a 317-image real validation split for computing the class-specific reference gradients used in synthetic-sample selection, and evaluated on the 200-image real test set used for the limited-data synthetic-augmentation experiments. Synthetic images were then added to the limited-data regime in progressively larger class-balanced budgets, as detailed in Section 4.2. The four detection classes for batteries are cylindrical, pouch, button, and other. The “other” class includes battery types that do not fall into the cylindrical, pouch, or button categories, such as lead-acid batteries and other irregular or less common battery form factors. This design makes it possible to assess not only whether synthetic augmentation helps, but also how much synthetic data is beneficial before diminishing returns appear.

#### 4.1.1 Real X-ray dataset

**Figure 6:**
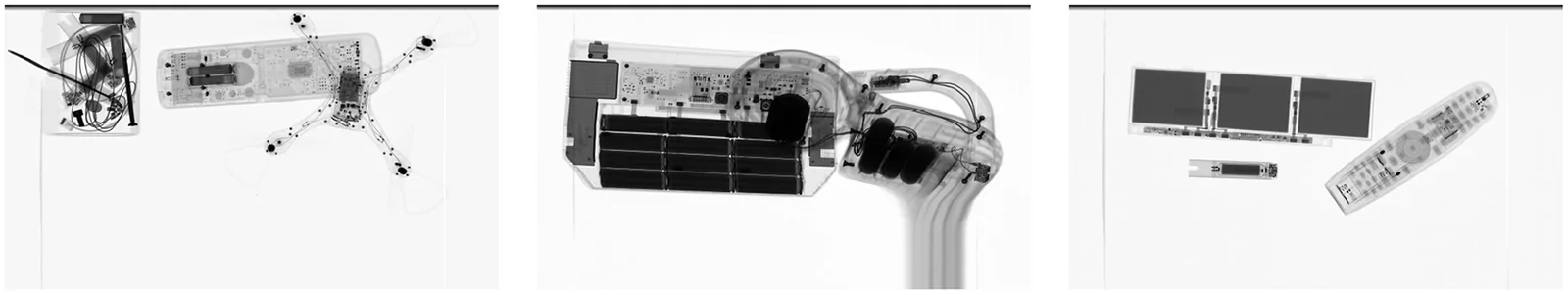
Representative real X-ray images from the WEEE dataset. The examples show the diversity of scanned devices, including cluttered assemblies, overlapping electronic components, cylindrical battery packs.

The real-world dataset consisted of 127 scanned WEEE devices, producing 917 distinct X-ray images. Dataset splits were performed at the device level to prevent overlap between training, validation, and test sets. The scanned devices were acquired from WEEE recycling bins and primarily included smartphones, tablets, small vacuum cleaners, battery packs, and electronic cigarettes. Example real X-ray images from the dataset are shown in Figure 6. A complete breakdown of the device, image, and per-class object instance counts for the training, validation, and test partitions of both experimental regimes is provided in Table 1. The dataset is dominated by cylindrical cells, while the “other” category is comparatively rare, reflecting the natural class distribution of batteries encountered in the WEEE stream.

**Table 1:** Splits were performed at the device level to prevent overlap between partitions.

Regime	Split	Devices	Images	Object instances per class				
				Cyl.	Pouch	Button	Other	Total
Full-data	Training	111	800	2,960	879	241	38	4,118
	Test	16	117	433	128	36	6	603
	**Total**	**127**	**917**	**3,393**	**1,007**	**277**	**44**	**4,721**
Limited-data	Training	55	400	1,480	439	121	19	2,059
	Validation	44	317	1,173	348	96	15	1,632
	Test	28	200	740	220	60	10	1,030
	**Total**	**127**	**917**	**3,393**	**1,007**	**277**	**44**	**4,721**

Instances are reported by class: cylindrical (Cyl.), pouch, button, and other. The full-data regime used 800 training and 117 test images with no validation split or synthetic-sample ranking. In the limited-data regime, the validation set was used to compute class-specific reference gradients for synthetic-sample selection. Bold values indicate the totals across all partitions within each experimental regime.

The scanning phase was performed on an X-Ray scanning machine with 0.1 m/s scanning speed. The scanning machine utilized a Dual Energy X-Ray detector. The result of each scan was a set of two 1280 × 960 images, one for Total Energy and one for High Energy. Total Energy value gives the number of all photons captured by the detector for each pixel, while High Energy value gives the number of photons captured above an Energy Threshold (30 keV).

#### 4.1.2 Synthetic datasets

To evaluate the contribution of both domain adaptation and subset selection, we considered four augmentation settings under the same real-data proportions, gradient-selected Blender synthetic images, randomly sampled Blender synthetic images, gradient-selected CycleGAN synthetic images, and randomly sampled CycleGAN synthetic images. Within the limited-data synthetic-augmentation experiments, the same 200-image held-out real test set was used for every condition. The full-data 800-image real-only baseline was evaluated separately on a 117-image held-out real test set. Fixed test sets are particularly important within each experimental regime because they prevent performance differences from being attributed to different evaluation splits.

For each synthetic-augmentation condition, candidate synthetic images were ranked before detector training using the class-conditional gradient-alignment criterion described in Section 3.2. The top-*K* synthetic images per class were added to the corresponding real training split. We evaluated *K* ∈ {0, 10, 20, 25, 30, 40, 50} synthetic images per class, corresponding to total synthetic augmentation sizes of 0, 40, 80, 100, 120, 160, and 200 images across the four battery classes. To evaluate the effect of selection independently from the number of synthetic images added, each gradient-selected condition was paired with a random-selection baseline using the same value of *K* and the same total number of added synthetic images. This comparison was performed for both Blender-only synthetic images and CycleGAN-translated synthetic images, enabling fair comparisons of sample selection and synthetic-to-real translation under matched synthetic image counts.

### 4.2 Implementation details

All detection experiments were performed using the YOLOv8-m object detector within a fixed training protocol so that differences in performance could be attributed to the proposed synthetic-data pipeline rather than changes in detector architecture or optimization strategy. The same detector was used for all experimental conditions, while only the amount of real training data and the composition of the injected synthetic data were varied. YOLOv8-m models were trained for 100 epochs with a batch size of 8 and an input image resolution of 1, 280 × 960 pixels. These settings were held constant across all baselines and augmentation conditions to ensure fair comparison between real-only training, Blender-based synthetic augmentation, and CycleGAN-translated synthetic augmentation.

Moreover, all images and annotations were converted to the standard YOLO detection format, with one annotation file per image containing normalized bounding-box coordinates and class labels. TE and HE images were used as stacked three-channel representation. The training, validation, and test partitions were kept fixed for all experiments, and the held-out real test set was never used during model selection or synthetic-data ranking. In addition, the real validation split used to compute class-specific reference gradients for synthetic ranking and was kept separate from the final detector test set, minimizing information leakage between data selection and final evaluation. This separation is consistent with the methodological design introduced in Section 3.2, where gradient-based selection is performed prior to detector training using a held-out real validation subset.

For experiments involving synthetic augmentation, candidate synthetic images were first generated in Blender and then optionally translated to the real-image domain using CycleGAN before ranking. The CycleGAN training protocol used synthetic and real images (from Section 3.1.6 for training, with optimization based on Adam, a learning rate of 2 × 10^−4^, β_1_ = 0.5, 100 epochs at constant learning rate, and 100 epochs with linear decay to zero, following the standard CycleGAN training regime. The translated synthetic images produced by this generator were then treated as an alternative synthetic pool for downstream selection and YOLO training. CycleGAN's unpaired translation framework and the LSGAN objective used in this work follow the original formulation of (Zhu et al. 2017).

Model performance was evaluated on the held-out real test set using mAP50 and mAP50:95. The latter metric averages detection performance across IoU thresholds from 0.50 to 0.95 and provides a stricter assessment of localization accuracy than mAP50 alone, making it especially useful for comparing improvements under limited labeled data. This follows the standard evaluation practice used in modern YOLO pipelines and COCO-style object detection benchmarking. All experiments were conducted in Python 3.10 using PyTorch 2.1.2, Ultralytics 8.0.196, and CUDA 11.8 on an NVIDIA RTX 3090 GPU.

Each detector training was repeated across three independent trials with different random seeds affecting model initialization, data shuffling, and random synthetic sampling. For gradient-selected conditions, the same ranking procedure was applied for each trial using the corresponding training setup. Unless otherwise stated, results are reported as mean ± standard deviation across the three trials.

### 4.3 Results

**Figure 7:**
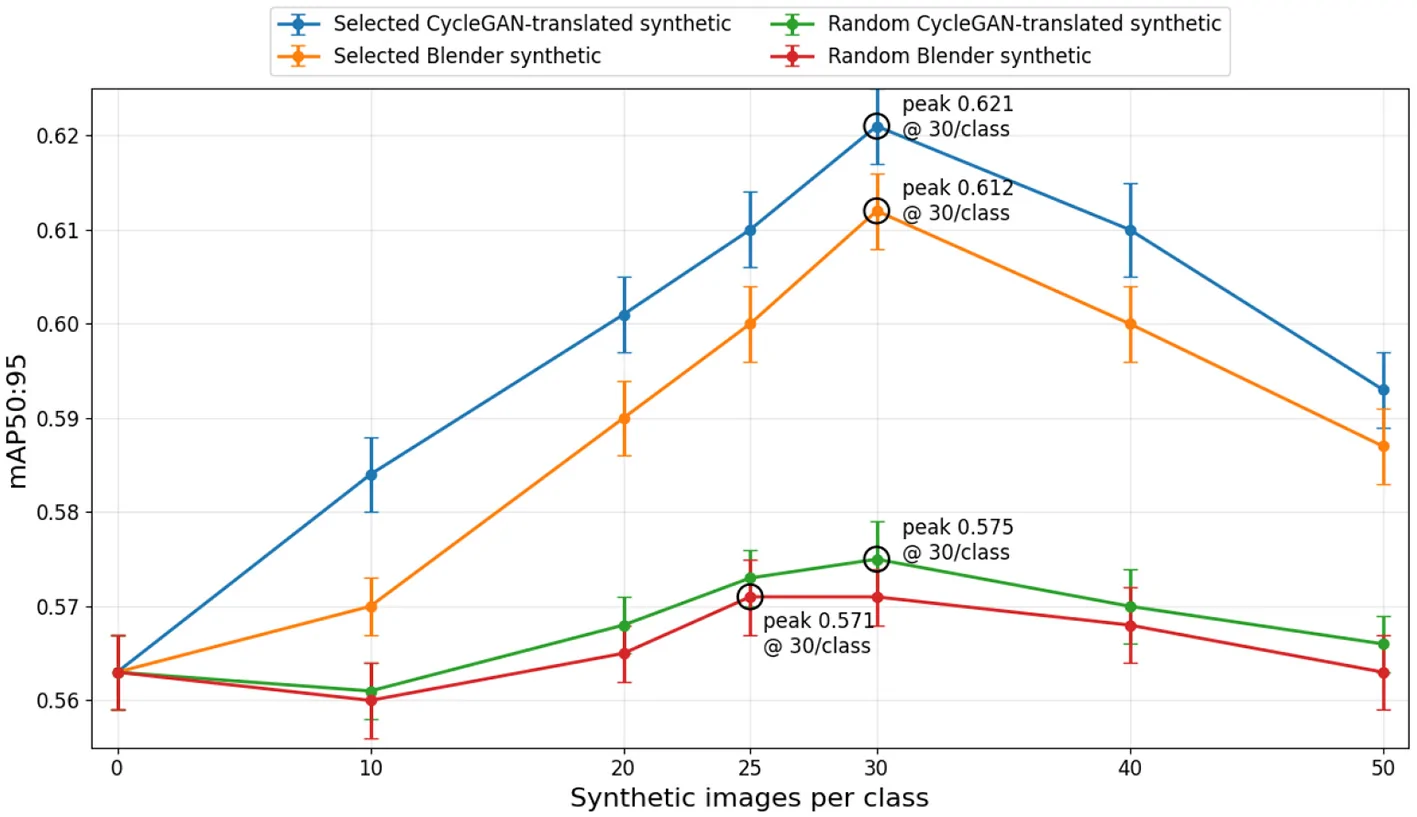
Detection performance as a function of synthetic budget under limited labeled data.

As summarized in Table 2 and visualized in Figure 7, synthetic augmentation was not uniformly beneficial at all injection levels, and gradient-selected subsets consistently outperformed random subsets under matched synthetic budgets. Performance improved as the synthetic budget increased from +10 to +30 synthetic images per class, but gains saturated beyond this point and then slightly declined at the largest budgets. For the selected CycleGAN-translated synthetic setting, mean mAP50:95 increased from 0.584 ± 0.004 at +10 synthetic images per class to 0.601 ± 0.004 at +20, 0.610 ± 0.004 at +25, and 0.621 ± 0.004 at +30, before decreasing to 0.610 ± 0.005 at +40 and 0.593 ± 0.004 at +50. A similar but slightly weaker pattern was observed for selected Blender-only synthetic images, which increased from 0.570 ± 0.003 at +10 per class to 0.612 ± 0.004 at +30 per class and then declined thereafter. This trend indicates that the proposed method benefits from moderate, targeted synthetic augmentation, rather than simply maximizing the number of synthetic samples added to the training set.

**Table 2:** Effect of increasing synthetic augmentation size under the limited-data setting using 400 real training images.

Synthetic images per class	Selected CycleGAN mAP50:95	Selected Blender mAP50:95	Random CycleGAN mAP50:95	Random Blender mAP50:95
0	0.563 ± 0.004	0.563 ± 0.004	0.563 ± 0.004	0.563 ± 0.004
10	0.584 ± 0.004	0.570 ± 0.003	0.561 ± 0.003	0.560 ± 0.004
20	0.601 ± 0.004	0.590 ± 0.004	0.568 ± 0.003	0.565 ± 0.003
25	0.610 ± 0.004	0.600 ± 0.004	0.573 ± 0.003	**0.571** **±0.004**
30	**0.621** **±0.004**	**0.612** **±0.004**	**0.575** **±0.004**	**0.571** **±0.003**
40	0.610 ± 0.005	0.600 ± 0.004	0.570 ± 0.004	0.568 ± 0.004
50	0.593 ± 0.004	0.587 ± 0.004	0.566 ± 0.003	0.563 ± 0.004

Results are reported as mean ± standard deviation over three trials. Bold values indicate the highest mean mAP50:95 achieved within each column. In the Random Blender column, the same highest mean value was obtained at two synthetic budgets (+25 and +30 images per class) and both are therefore shown in bold.

The main result of this study, based on Table 3, is that the proposed synthetic-data pipeline is most beneficial when labeled real X-ray data are limited. When the real-data budget was reduced to 400 images (100 per class), performance dropped to 0.563 mAP50:95 and 0.832 mAP50, confirming that detector accuracy degrades in the intended low-data regime. However, under this constrained setting, adding gradient-selected synthetic data consistently improved detection performance, with the strongest configuration obtained using domain-translated Blender synthetic data selected by the proposed gradient-based criterion. At a budget of +30 synthetic images per class (120 total synthetic images), this setting achieved 0.621 ± 0.004 mAP50:95 and 0.864 ± 0.003 mAP50, corresponding to an absolute improvement of +0.058 mAP50:95 and +0.032 mAP50 over the low-data real-only baseline. Notably, this best low-data configuration reached a numerically higher mAP than the larger 800-image real-only reference setting. We emphasize, however, that the two regimes were evaluated on different held-out test sets, 117 images for the 800-image full-data baseline and 200 images for the 400-image limited-data experiments, so this comparison should be read as indicative context rather than a direct superiority claim. The controlled, directly comparable finding is that, within the limited-data protocol and on a common test set, gradient-selected synthetic augmentation substantially outperformed both the real-only baseline and random synthetic sampling.

**Table 3:** Main detector results under full-data and limited-data experiments.

Real images	Synthetic augmentation	Synthetic images	mAP50:95	mAP50
800 (200/class)	None	0	0.590 ± 0.003	0.850 ± 0.003
400 (100/class)	None	0	0.563 ± 0.004	0.832 ± 0.003
400 (100/class)	Selected CycleGAN-translated Blender synthetic	120 (30/class)	**0.621** **±0.004**	**0.864** **±0.003**
400 (100/class)	Selected Blender synthetic	120 (30/class)	0.612 ± 0.004	0.855 ± 0.003
400 (100/class)	Random CycleGAN-translated Blender synthetic	120 (30/class)	0.575 ± 0.004	0.843 ± 0.003
400 (100/class)	Random Blender synthetic	120 (30/class)	0.571 ± 0.003	0.838 ± 0.004

The 800-image full-data baseline was evaluated on a separate 117-image held-out test set and is included as a reference point only; all limited-data conditions (400 real images) share a common 200-image held-out test set and are directly comparable to one another. Bold values indicate the best result among the limited-data (400 real image) conditions, which share a common held-out test set.

**Figure 8:**
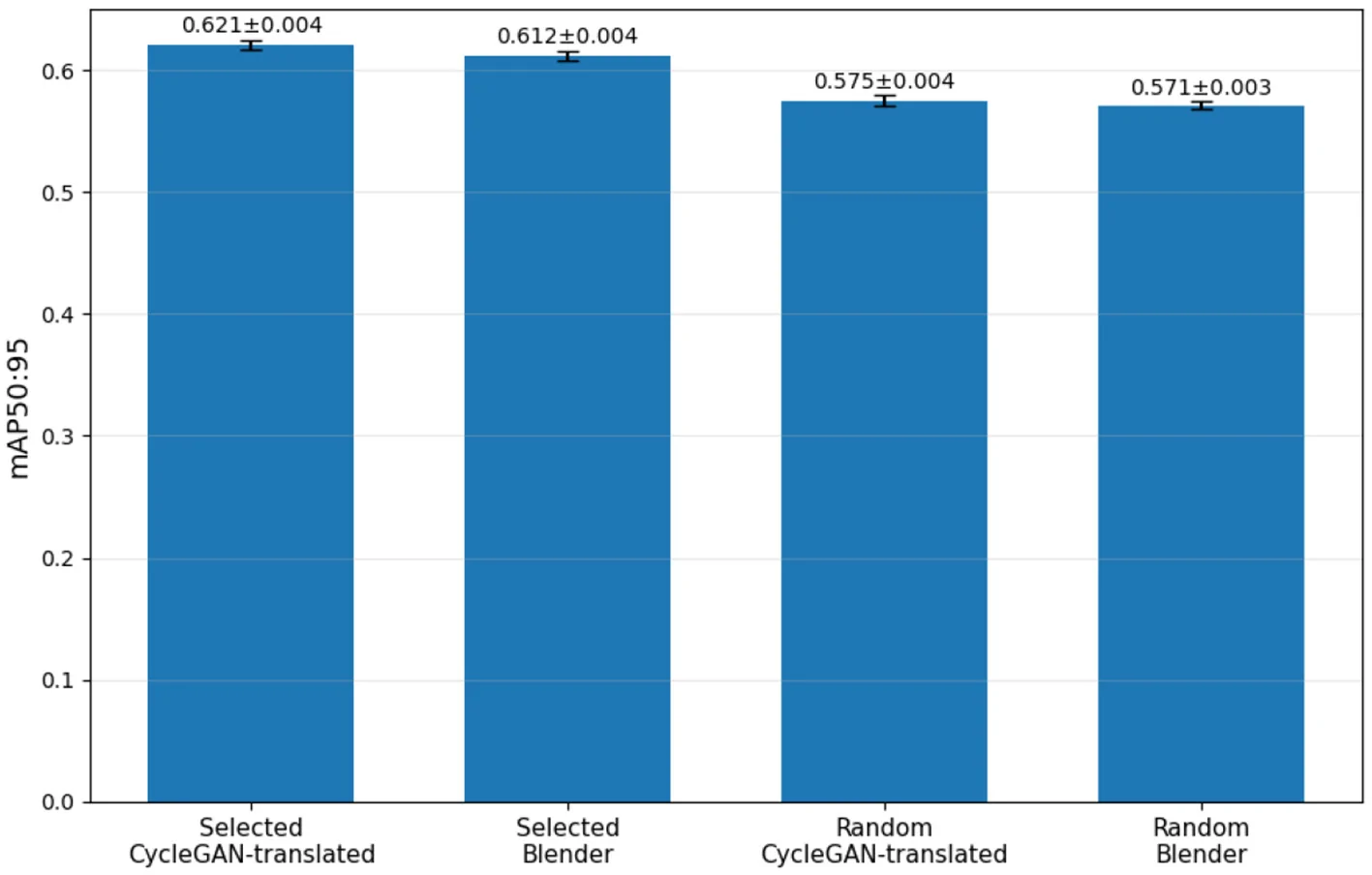
Effect of gradient-based selection vs. random sampling at +30 synthetic images per class. Bars show mean mAP50:95 over three trials, and error bars indicate standard deviation.

The +30/class comparison in Figure 8 further shows that sample selection quality matters more than synthetic quantity alone. At the strongest operating point of +30 synthetic images per class, gradient-selected CycleGAN-translated images achieved 0.621 ± 0.004 mAP50:95/0.864 ± 0.003 mAP50, whereas randomly sampled translated synthetic images reached only 0.575 ± 0.004 mAP50:95/0.843 ± 0.003 mAP50 under the same budget. Likewise, gradient-selected Blender synthetic images achieved 0.612 ± 0.004 mAP50:95/0.855 ± 0.003 mAP50, compared with 0.571 ± 0.003 mAP50:95/0.838 ± 0.004 mAP50 for random Blender sampling. These comparisons demonstrate that the performance gains cannot be explained solely by adding more synthetic data; rather, they arise from selecting synthetic samples whose learning signal is better aligned with real validation data. The strongest performance was obtained when domain adaptation and gradient-based selection were combined, suggesting that the two components play complementary roles, CycleGAN improves appearance realism, while gradient-based ranking improves sample relevance.

To further examine whether the best-performing configuration improved detection uniformly across battery categories, we report per-class AP for the selected CycleGAN-translated synthetic setting at +30 images per class. As shown in Table 4, performance was strongest for cylindrical and pouch batteries, which generally have larger and more regular X-ray appearances, while button and other battery categories remained more challenging due to smaller object size, greater shape variability, and stronger visual similarity to surrounding electronic components.

**Table 4:** Per-class AP for the selected CycleGAN-translated synthetic model at +30 synthetic images per class.

Class	AP50:95	AP50
Cylindrical	0.690	0.910
Pouch	0.660	0.890
Button	0.560	0.820
Other	0.574	0.836
**mAP**	**0.621**	**0.864**

Bold values in the final row indicate the mean average precision across all four battery classes; the preceding rows report per-class values.

**Figure 9:**
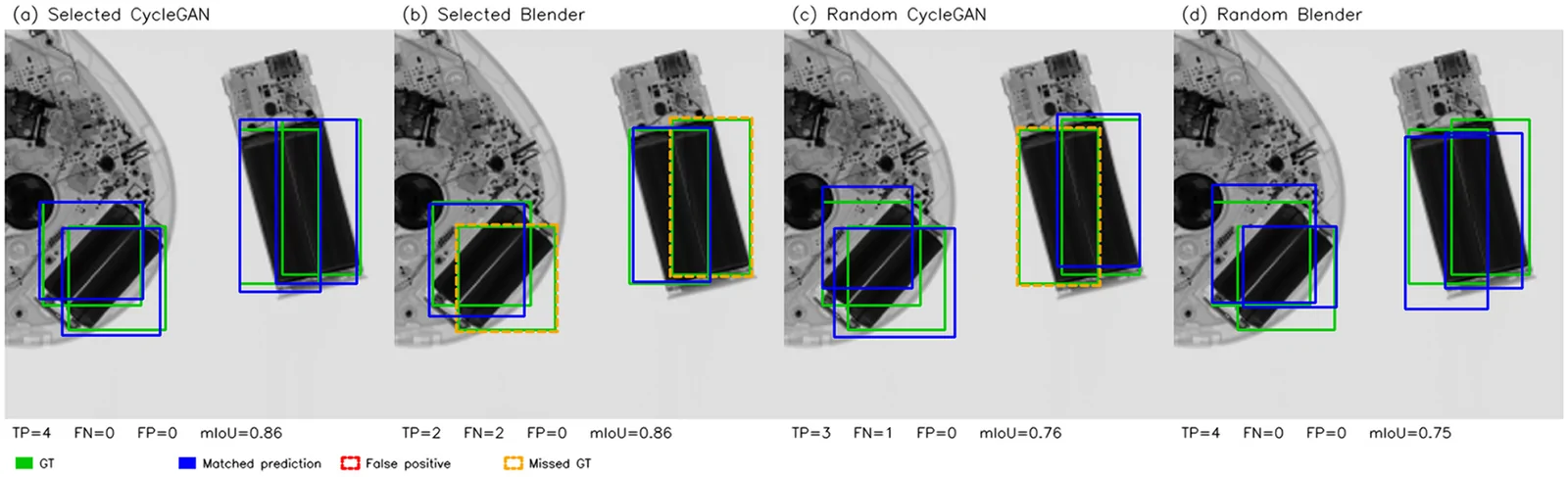
Qualitative detection comparison on a held-out real X-ray test image at +30 synthetic images per class. Green boxes denote ground truth, blue boxes matched predictions, and orange dashed boxes missed ground-truth objects

Figure 9 provides a qualitative example on a held-out real X-ray test image, comparing ground truth annotations with predictions from all four augmentation strategies at the +30 synthetic images per class setting.

### 4.4 Ablation study

To isolate the contributions of synthetic-to-real translation and gradient-based selection, we compared selected and random subsets for both Blender only and CycleGAN-translated synthetic data under identical low-data conditions, with the strongest +30/class operating point summarized in Table 5. The gap was especially clear at the most effective operating points. For example, at +20 synthetic images per class, the selected translated setting achieved 0.601 ± 0.004 mAP50:95, compared with 0.568 ± 0.003 for random translated sampling. At +25 per class, the corresponding values were 0.610 ± 0.004 vs. 0.573 ± 0.003, and at +30 per class the gap widened further to 0.621 ± 0.004 vs. 0.575 ± 0.004. Similar gains were observed for Blender-only synthetic images, where selected samples consistently outperformed random ones at the same budget. These findings support the hypothesis that synthetic augmentation is most effective when the augmented samples are not only realistic, but also informative with respect to the detector's gradient dynamics on real data.

**Table 5:** Ablation at the strongest low-data operating point, +30 synthetic images per class.

Method	mAP50:95	mAP50	Δ mAP50:95 vs. real-only
Selected CycleGAN-translated Blender synthetic	**0.621** **±0.004**	**0.864** **±0.003**	**+0.058**
Selected Blender synthetic	0.612 ± 0.004	0.855 ± 0.003	+0.049
Random CycleGAN-translated Blender synthetic	0.575 ± 0.004	0.843 ± 0.003	+0.012
Random Blender synthetic	0.571 ± 0.003	0.838 ± 0.004	+0.008

Δ indicates absolute mAP50:95 improvement over the 400-image real only baseline. Bold values indicate the best-performing configuration for each metric.

The ablation also clarifies the role of domain translation. Under matched gradient-based selection, CycleGAN-translated synthetic images consistently performed slightly better than raw Blender renders across the most relevant synthetic budgets. At +20 synthetic images per class, the selected translated setting achieved 0.601 mAP50:95, compared with 0.590 for selected Blender only synthetic images. At +25 per class, the corresponding results were 0.610 and 0.600, and at +30 per class they were 0.621 and 0.612, respectively. Although these gains were smaller than the selection vs. random gap, they remained consistent, indicating that domain translation provides an additional performance benefit by reducing the appearance gap between rendered and real X-ray images. The best overall detector performance was therefore achieved by combining Blender based generation, CycleGAN based translation, and gradient based subset selection in one pipeline.

## 5 Conclusion and discussion

This study investigated whether carefully curated synthetic data can improve battery detection in X-ray images when labeled real data are limited. We proposed a three-stage pipeline that combines Blender-based synthetic image generation, CycleGAN-based synthetic-to-real translation, and gradient-based subset selection before YOLOv8 training. Unlike naive augmentation, the proposed framework was designed to rank synthetic candidates by their alignment with gradients computed from held-out real validation data, enabling the model to prioritize synthetic samples that are both realistic and informative. The overall experimental design also controlled for confounding factors by using fixed detector settings, matched synthetic budgets, and a common held-out real test set across all augmentation conditions.

The results support the central hypothesis of the paper, that synthetic augmentation is most valuable in cases where labeled data are limited, and its benefit depends strongly on how the synthetic data is selected. The validated finding of this study is that, under the limited-data protocol and on a common held-out test set, gradient-selected synthetic augmentation improved YOLOv8-m detection compared with both the real-only baseline and random synthetic sampling baselines. In the low-data setting, the real-only detector achieved 0.563 mAP50:95 and 0.832 mAP50, whereas the best-performing configuration was CycleGAN-translated synthetic data, with gradient selection added at a moderate budget of 30 synthetic images per class, delivering improved performance to 0.621 mAP50:95 and 0.864 mAP50. The experiments also showed that gradient selection method outperformed random sampling under matched budgets, while CycleGAN-based translation provided an additional improvement over raw Blender renders.

These findings suggest that the most effective way to use synthetic data for low-data X-ray battery detection is not to maximize synthetic quantity, but to combine domain-adapted generation with task-aware sample selection. From an application perspective, this is encouraging for automated WEEE inspection, where collecting and annotating large real datasets is expensive, hazardous, and operationally difficult. Nevertheless, this study also has limitations. The experiments were conducted under a controlled benchmark with a fixed detector and a specific synthetic generation pipeline, and the conclusions should therefore be validated more broadly. Future work will proceed along four specific directions. First, external validation on independent WEEE X-ray datasets, such as publicly available battery detection benchmarks collected at other facilities, is needed to establish that the gradient-selection benefit transfers across acquisition systems, device distributions, and annotation protocols. Second, the pipeline should be tested with additional detector architectures beyond YOLOv8-m, including two-stage and transformer-based detectors, to verify that the selection criterion is not specific to a single detection head design. Third, extending the framework to segmentation-aware models, such as instance segmentation architectures that exploit the pixel-level masks already produced by our synthetic pipeline, would enable finer-grained localization of battery boundaries. Finally, the pipeline should be evaluated within robotic extraction workflows, where detection outputs drive automated battery removal, in order to quantify how the observed mAP improvements translate into end-to-end sorting throughput and safety gains in operational recycling lines.

## Figures and Tables

**Table:** 

Symbol	Description
*I*(ω)	Attenuated X-ray intensity at photon energy ω
*I*_0_(ω)	Initial (unattenuated) intensity spectrum of the X-ray source
*x*	Distance traversed by a ray inside a material
*p*	Ray–mesh intersection point along a cast ray
*S*	Synthetic image domain (Blender-generated X-ray images)
*R*	Real image domain (scanned X-ray images)
*G*	CycleGAN generator mapping the synthetic domain to the real domain, *G*:*S*→*R*
*F*	CycleGAN generator mapping the real domain to the synthetic domain, *F*:*R*→*S*
*c*	Battery class index (cylindrical, pouch, button, other)
*i*	Index of a synthetic candidate image
*g*_*c*_	Reference gradient computed from real validation images of class *c*
*g*_*i*_	Detection-loss gradient of synthetic candidate *i* on the trainable detection head
align(*i*)	Non-negative clipped cosine gradient-alignment score of candidate *i*
*K*	Number of gradient-selected synthetic images per class

**Table:** 

Symbol	Description
ω	X-ray photon energy level
μ(ω)	Energy-dependent linear attenuation coefficient of a material
β_1_	Exponential decay rate of the first-moment estimate in the Adam optimizer

**Table:** 

Index	Description
0	Initial (source) quantity before attenuation, as in *I*_0_(ω)
*c*	Class-conditional quantity, as in the reference gradient *g*_*c*_
*i*	Per-candidate quantity, as in the candidate gradient *g*_*i*_
1	First-moment estimate of the Adam optimizer, as in β_1_
2*A*, 2*B*, 2*C*	Ray index and segment label of a ray-traversal segment, as in *x*_2*B*_ (Figure 4)
2*a*, 2*b*, 2*c*	Ray index and intersection-point label, as in *p*_2*b*_ (Figure 4)

## Data Availability

The datasets presented in this article are not readily available because the company of the detector has not agreed on publicing the total dataset. Requests to access the datasets should be directed to Myrto Inglezou, mi23878@essex.ac.uk.
